# Animal Combustion

**Published:** 1882-04

**Authors:** 


### ANIMAL COMBUSTION.

WITHIN every living organism there are two opposing forces. The "vital force," which produces all the phenomena of life, holds the material elements in unstable relations, against their will, so to speak, and it is antagonized by the natural chemical affinities of the elements, which tend to break down the organic compounds and re-arrange the elements in more stable form. This decomposition takes place in some degree during the life of every organism, and when life ends, or when the vital force ceases to act, it rapidly destroys the structure.

The waste matter resulting from this disintegration must be immediately removed from the body of the living animal, otherwise it clogs and poisons the system. The method of its accomplishment is one of the most admirable functions of the animal economy. To remove the effete matter in the natural liquid or solid state would be very exhausting; consequently it is burned, and the gaseous products of its union with oxygen are then easily carried away. Literally speaking, this makes a furnace of the body of every animal; and the most pressing and ceaseless demand of the system is for oxygen to support its fires. Respiration is hence an absorbing and excreting process, whereby oxygen is received and carbonic acid and water removed. It thus becomes a measure of the amount of combustion.

In the "cold-blooded" animals, respiration bears a direct proportion to the activity and the heat of the body, as the former causes a metamorphosis and waste of tissue, and the latter always aids decomposition. The fact is one of common observation. It is well illustrated in the quickened breathing of a tired animal, and in the almost entire suspension of respiration in the hibernating state. The respiration of a "cold-blooded" creature is increased by artificial heat. In extremely hot weather, frogs may have to leave the water entirely, and fishes come to the surface to procure air. Reversely, frogs, can be kept for years in a state of suspended animation by a low temperature, and revived by warming. Some low animals can survive freezing or drying for an indefinite time; and, under such conditions, the waste of the tissues must be entirely suspended.—"*How Animals Breathe*," *in Popular Science Monthly.*

---

THERE is no department of the Federal government where, from the nature of the business, there are such opportunities for frauds as in the pension bureau. There are already 700,000 names registered of pensions. There are 130,000 other applicants whose cases have not been decided, and new cases are coming in at the rate of 100 a day. It would be naturally supposed that as time passes the number of pensioners would be decreased by death, but such is not the case. The expenditures in 1882 for pensions is estimated at $88,000,000, and it is supposed that between $10,000,000 and $20,000,000 of this amount will be on fraudulent claims.

### ALL ABOUT HORSES.

### HOW TO BUY A HORSE.

IN buying a horse, says the *Turf, Field and Farm*, look first to his head and eyes for signs of intelligence, temper, courage and honesty. Unless a horse has brains you cannot teach him to do anything well. If bad qualities predominate in a horse, education only serves to enlarge and intensify them. The head is the indicator of disposition in a horse. A square muzzle with large nostrils evidences an ample breathing apparatus and great lung power. Next, see that he is well cut under the jowl, with jaw bones broad and wide apart under the throttle. Breadth and fullness between the ears and eyes is always desirable. The eye should be full aud hazel in color, ears small and thin and thrown well forward. The horse that turns his ears back until they almost meet at the point is not to be trusted. He is a biter or a kicker, and is sure to be vicious in other respects. A horse with a dishing face is cowardly, and a cowardly brute being naturally vicious can never be trained to anything well. A rounding nose, tapering forehead and a broad, full face below the eyes is always mischievous and treacherous. Avoid a long-legged, stilty horse. Select one with a short, straight back and rump, withers high and sloping, well set back and broad, with good depth of chest, fore legs short, hind legs straight with the hock low down, short pastern joints, and a round mulish foot. By observing the above hints a horse may be selected that is graceful, good-natured, serviceable and a prize to the owner.

### ABOUT WATERING HORSES.

Horses need much more water during the day and at night than most persons suppose. When a horse needs water, if he does not receive the needed supply, we have no idea of the intense suffering which the poor creature must endure. After a horse has been driven until he prespires profusely, there will be an imperative demand for water to supply the place of the liquid that has passed off through the pores of the skin; and, after a horse has filled his stomach witn dry feed, a little water is needed to promote digestion, especially when the animal did not receive a generous supply before he was fed. When the stomach and bowels need more water they will have it, if the supply must be taken out of the skin. But when the digestive organs must draw extensively on water that is secreted in the tissues of the flesh and muscles, we cannot compute the great injury that must follow such an unnatural way of obtaining a supply of water, which is absolutely needed to promote healthful and complete digestion.

The digestive organs cannot perform their proper functions without water any more than a fire can be made without wood or coal. As the stomach of a horse is exceedingly small when compared with the first stomach or rumen of meat cattle, we perceive the vast importance of supplying a little water and often, rather than permit the thirsty brute to swallow several gallons at one draught only once or twice during twenty-four hours. During a period of more than fifty years past I have taken personal care of horses, have owned and reared horses, and have never had a sick horse or one injured or disabled. My rule is now, and ever has been, to water, feed and take good care of my horses before I seek refreshments and comfort for myself. When horses are watered frequently they will drink only a few quarts at each draught. This is infinitely better than to allow them to gulp down at one draught two or three pailfuls. It is better to let a horse drink at least a pailful before eating than to drink copiously after his meal. A large quantity of water after feeding will often drive much of the feed from the stomach before it is half digested.—*S. E. T. in Evangelist.*

